# Scientific evaluation of design and surface advances in Straumann implants

**DOI:** 10.4317/medoral.27841

**Published:** 2026-01-24

**Authors:** Pablo Cea-Arestín

**Affiliations:** 1 Department of Surgery and Medical-Surgical Specialties, School of Medicine and Dentistry, University of Santiago de Compostela, Santiago de Compostela, Galicia, Spain

## Abstract

**Background:**

Contemporary dental implant systems are continuously evolving in terms of surface characteristics, as well as macro- and micro-design. This study evaluates the performance of recent Straumann® implants-specifically comparing Bone Level Tapered (BLT) and Bone Level Conical (BLC) designs-in relation to peri-implant bone resorption and immediate loading protocols.

**Material and Methods:**

A total of 84 BLT implants and 84 BLC implants were placed in 78 patients between 2023 and 2025 across several clinics in Spain. Radiographic and periodontal probe measurements were performed to assess crestal bone level changes at one year post-placement. This retrospective cohort study focused on primary outcomes including 1-year survival rates and the evaluation of marginal bone loss (MBL).

**Results:**

The most significant result was the 98.8% success rate observed for BLC implants.

**Conclusions:**

While both Straumann® tapered implants represent excellent treatment options, BLC implants demonstrated superior improvements across all evaluated parameters. Future research should focus on identifying areas for further enhancement and comparing their performance with other leading implant systems.

## Introduction

This study evaluates the performance of recent Straumann® implants-specifically comparing Bone Level Tapered (BLT) and Bone Level Conical (BLC) implants design-in relation to peri-implant bone resorption and immediate loading protocols.

Contemporary dental implant systems are continuously evolving in terms of surface characteristics and macro- and micro-design. These developments aim to enhance osseointegration speed, minimize marginal bone loss, enable immediate loading protocols and improve overall implant survival rates (1).

Straumann® is recognized as a global leader in implant dentistry, with over seven decades of clinical innovation, as it was the pioneer in introducing both the titanium-zirconium alloy Roxolid® and the hydrophilic SLActive® surface (2 , 3).

The BLT implant, a representative of tapered implant systems, features a body design characterized by concentric steps that progressively narrow toward the apex, a configuration designed to reduce surgical invasiveness and enhance primary stability (4). Scientific literature supports the fact that tapered implants, such as BLT, provide superior primary stability compared to cylindrical counterparts, particularly in low-density bone or post-extraction sites (5).

The BLC implant system represents a new generation of tapered implants, optimized for bone-level placement. The design includes an ultra-narrow apex for an improved fit in anatomically constrained sites such as narrow ridges, converging root tips, or facial cavities, easing minimally invasive procedures while providing robust primary and secondary stability even in challenging bone conditions (6).

Common characteristics between these systems include the SLActive® surface and Roxolid® alloy, which contribute to higher survival rates due to their better biocompatibility and biomechanical properties (2 , 3). However, their design differences influence clinical handling and specific indications. This comparative assessment highlights how innovation in implant macro-geometry and prosthetic integration can optimize performance under immediate or delayed loading conditions, particularly in anatomically or biologically challenging cases.

## Material and Methods

This retrospective cohort study was conducted by reviewing the clinical and radiographic records of patients treated between 2023 and 2025 across several clinics in Spain. A total of 84 BLT implants and 84 BLC implants were placed in 78 patients, following a standardized drilling protocol and positioning the implants 1 mm subcrestally. Radiographic and periodontal probe measurements (7) were performed to assess crestal bone level changes at one year post-placement. Implants were loaded at 4 months post-placement in the maxilla and 3 months in the mandible for the BLT group, and at 3 and 2 months, respectively, for the BLC group, in accordance with reported loading protocols in the literature (8). The primary outcomes assessed were 1-year survival and marginal bone loss (MBL).

The inclusion criteria encompassed patients aged 25 years or older who had received BLT and/or BLC implants, regardless of chronic medication or underlying systemic diseases. To minimize potential bias, the following baseline data were collected: Gender, age, arch (maxilla/mandible), site (anterior/posterior), gingival phenotype, bone quality, and history of periodontitis.

The variables considered in the study included implant type (BLT or BLC), success/failure, post-extraction versus healed sites, immediate versus delayed loading, use of a cover screw or healing abutment, the performance of simultaneous bone and/or soft tissue grafting, and MBL.

Radiographs were obtained using a standardized parallelization technique with individualized positioning devices to ensure reproducible angulation. MBL was measured vertically from the most coronal point of the bone crest to the implant shoulder. Calibration was performed using known implant dimensions as a reference to correct for potential magnification errors. Measurements were carried out under optical magnification to enhance precision and reproducibility.

Independent observers performed all measurements. Inter-observer calibration was conducted using 20 randomly selected radiographs, repeated after a 2-week interval. Agreement between observers was assessed by the Intraclass Correlation Coefficient (ICC) based on a two-way random-effects model with absolute agreement. The ICC value was 0.78, indicating good reliability according to the Landis and Koch criteria (9). This level of reliability is consistent with similar radiographic assessments reported in implant studies, supporting the validity of the present measurements (10). Distribution analyses according to site and arch were performed.

Continuous variables (MBL) were expressed as mean $\pm$ standard deviation (SD). Normality was assessed using the Shapiro-Wilk test. Since the groups showed unequal variances, Welch's t-test was employed to compare MBL between BLT and BLC implants. Categorical variables (implant failure, location) were analyzed using Fisher's Exact Test to determine Odds Ratios (OR) and Relative Risk (RR) with a 95% confidence interval. Statistical significance was set at p&lt;0.05. All analyses were performed using Microsoft Excel.

## Results

Global results are summarized in Table 1. A Welch's t-test revealed a significant difference in MBL between the groups (t \approx 17.7, p&lt;0.0001).

Table 1

Table 2 examines the specific circumstances associated with implant failures. Analysis using Fisher's Exact Test yielded an Odds Ratio of 6.38 (p=0.116) and a Relative Risk (RR) of 6.0.

Table 2

Mean MBL (±SD) was 0.67±0.14mm for the BLT group and 0.23±0.18mm for the BLC group. Distribution analysis according to site and arch showed that MBL was generally higher in mandibular and posterior sites compared to maxillary and anterior regions, consistent with patterns of biomechanical load distribution. Specifically, 46.4% of implants in the BLT group and 56.0% in the BLC group were located in the maxilla, while posterior teeth represented 72.6% and 63.1% of the sites, respectively.

Table 3 presents the baseline data characteristics.

Table 3

## Discussion

As observed in the results, both implant types demonstrate excellent clinical performance; however, the BLC implant overall yielded superior outcomes, rendering it a preferred choice in most clinical scenarios.

There is a noticeable improvement in osseointegration success (11) associated with the BLC implant, regarding both short-term factors-such as immediate placement and loading (12)-and long-term outcomes, including the stabilization of MBL (13). Due to its enhanced connection design, the BLC implant promotes superior bone and soft tissue remodeling, which is crucial for preventing early MBL (14). Furthermore, its innovative geometry enhances primary stability across a wide range of clinical situations, potentially reducing the need to resort to alternative designs, such as BLX implants.

One consequence of the frequent use of BLC implants in post-extraction scenarios is a higher frequency of bone grafting procedures. However, this is counterbalanced by a reduced need for soft tissue grafts, as the implant's design and connection facilitate optimal esthetic and biological outcomes while minimizing biological, temporal, and financial risks.

Regarding implant failures, Fisher's Exact Test (Odds Ratio: 8.22; p=0.037) and the calculated Relative Risk (7.5) indicate a statistically significant difference in failure rates for post-extraction cases between the BLT and BLC groups (p&lt;0.05). These findings suggest that BLT implants placed in post-extraction sites are 7.5 times more likely to fail compared to BLC implants under the same clinical conditions. This highlights the importance of selecting the most appropriate implant design based on the timing of placement (15).

There are, however, inherent limitations to this research. Not all patients present the same bone quality or gingival phenotype, and adherence to maintenance regimens may vary. Implant placement sites differ, as do individual responses to grafting procedures and the prosthetic components produced by different laboratories. Nonetheless, efforts were made to minimize variability by following a standardized protocol for all cases. The limited follow-up time remains another factor to consider.

## Conclusions

Straumann's tapered implants constitute an excellent treatment option; however, the BLC design demonstrated superior improvements across all evaluated parameters. Future research should focus on identifying potential areas for further enhancement and comparing the performance of these designs with other leading implant systems.

## Figures and Tables

**Table 1:** Table Global results.

Variable	BLT (n=84)	BLC (n=84)
Success rate	78/84 (92.3%)	83/84 (98.8%)
Failure rate	6/84 (7.1%)	1/84 (1.2%)
Immediate placement	56/84 (66.7%)	75/84 (89.3%)
Immediate loading	43/84 (51.2%)	51/84 (60.7%)
Cover screw	14/84 (16.7%)	12/84 (14.3%)
Healing abutment	25/84 (29.8%)	21/84 (25.0%)
Bone graft	61/84 (72.6%)	79/84 (94.0%)
Gingival graft	12/84 (14.3%)	8/84 (9.5%)
Mean MBL (±SD)	0.67mm ± 0.14	0.23mm ± 0.18

1

**Table 2:** Table Specific circumstances associated with implant failures.

Group	Total failures	Postextraction sites	Immediate loading	Bone Graft
BLT (n=84)	6 (7.1%)	5/6 (83.3%)	5/6 (83.3%)	5/6 (83.3%)
BLC (n=84)	1 (1.2%)	1/1 (100%)	1/1 (100%)	1/1 (100%)

2

**Table 3:** Table Baseline data characteristics.

Baseline characteristics	BLT (n=84)	BLC (n=84)
Gender	49 men (58.3%) / 35 women (41.7%)	56 men (66.7%) / 28 women (33.3%)
Age (mean)	58.5 years	62.1 years
Maxilla / Mandible	39 maxilla (46.4%) / 45 mandible (53.6%)	47 maxilla (56.0%) / 37 mandible (44.0%)
Anterior / Posterior tooth	23 anterior (27.4%) / 61 posterior (72.6%)	31 anterior (36.9%) / 53 posterior (63.1%)
Gingival phenotype	41 thick (48.8%) / 33 thin (51.2%)	37 thick (44.0%) / 47 thin (56.0%)
Bone quality	45 soft (53.6%) / 39 hard (46.4%)	41 soft (48.8%) / 43 hard (51.2%)
Previous periodontitis	58 yes (69.0%) / 26 not (31.0%)	67 yes (79.8%) / 17 not (20.2%)

3

## Data Availability

The data sets used and/or analyzed during the current study are not available.
